# Chiropractors’ perception of occupational stress and its influencing factors: a qualitative study using responses to open-ended questions

**DOI:** 10.1186/s12998-016-0083-1

**Published:** 2016-02-22

**Authors:** Shawn Williams

**Affiliations:** CUNY York College – Department of Health Professions, 94 - 20 Guy R. Brewer Blvd, Jamaica, NY 11451 USA; Department of Research, New York Chiropractic College, Seneca Falls, NY USA; Private Practice - Montclair Performance Health & Chiropractic, LLC, Montclair, NJ USA

**Keywords:** Chiropractic, Psychological stress, Occupational stress, Qualitative research, Burnout

## Abstract

**Background:**

Job stress and emotional exhaustion have been shown to have a negative impact on the helping professional. The development and causal relations of job stress and emotional exhaustion are rather unclear in the chiropractic profession. The objective of this study is to understand the main sources of occupational stress and emotional exhaustion among doctors of chiropractic.

**Methods:**

Analysis of the written responses to web-based open-ended questionnaire was performed using an interpretive research methodology. Additionally, cross tabulation and Chi square statistical tests were conducted to match and couple the demographic data with the categorical themes.

**Results:**

Fourteen professional stress categories emerged from the 970 completed surveys. “Managed Care Organization regulation”, “Managed Care reimbursement” and “Scope of Practice Issues” were the most common stressors that negatively influenced chiropractors’ professional and personal lives. The results of the categorical analysis suggests that age, marital status, number of years in practice and location of practice may have an influence on the category of stress reported by chiropractors.

**Conclusions:**

The qualitative approach revealed common, conventional and culture-specific job stressors in doctors of chiropractic. Notably, these findings suggest an association between third-party payer influences (increased regulation/decreased reimbursement) with that of increased job stress. Further research will be undertaken to refine the stress and satisfaction parameters and address stress interventions.

## Background

Occupational stress and emotional exhaustion (EE) are extensive problems for health-care workers [1–4]. Occupational stress refers to environmental conditions and situations that prompt an emotional response such as anger or anxiety [5]. Similarly, but operationally and conceptually distinctive, EE is a chronic state of physical and emotional depletion that results from excessive job demands, depletion of resources and continuous hassles [6–9]. The relationship between occupational stress and EE is outlined in a collection of theoretical models, such as the control-stress model [5] and job-demands control model [10, 11]. The conceptual framework underlying occupational stress models [4, 10, 11] provide insight to the disputable relationship between job-demands, job resources and perception [5].

Accumulated research on occupational stress has generated a wealth of knowledge about the stress process and how stress affects workers in a wide variety of jobs [12–15]. McManus et al. [16] suggest that EE and occupational stress may have reciprocal causation – that is, high levels of EE may cause stress, and high levels of occupational stress caused may EE. Either way, the end-result of escalating occupational stressors in the health-care the arena is that the provider(s) experiences a shift from energy to exhaustion, engagement to cynicism and efficacy to infectiveness [1]. Similar processes have been observed in a comprehensive group of health professionals [2, 4, 17], and more recently in the chiropractic profession [18, 19]. A recent study [18] exploring burnout in the chiropractic profession suggests that although overall values of burnout are relatively low (~2 %), higher levels of EE (~21 %) remain workplace issues for this professional group.

As changes in the political sector of the health care arena continue to mount, and new socioeconomic trends occupy the environment of health care, the nature and construct of occupational stress and EE may continue to develop as a serious threat to workers’ well-being [5, 10, 20–23]. As such, despite being common in health-care workers [2–4, 17], the development and causal relations of occupational stress and EE are rather unclear in the chiropractic profession, in part due to an absence of adequate exploratory qualitative studies. Thus, the current study attempted to understand chiropractors’ perceptions of job-related stress and consequently EE (as per the symbiotic relationship that exist between the two constructs). The objective of this study was to explore the opinions and perceptions of occupational stress among Doctors of Chiropractic (DCs). In this study, the aim was to assess what reasons (if any) DCs give as their precursors of occupational stress. The use of qualitative methods to analyze the material derived from open-ended answers in a questionnaire was employed with the intent of creating occupational stress categories that could help increase an understanding of the phenomenon as it applies to the chiropractic profession. A greater understanding of the perceptions of occupational stress may, in turn, provide a means to understand and improve chiropractic services.

## Methods

A demographic survey including six socio-demographic categorical questions and one open-ended question was emailed (July 2013) to a randomized and convenience sample of DCs whose email addresses were included in the database of a leading chiropractic-marketing agency [24]. The invitation letter included a description of the nature of the study, a notation guaranteeing anonymity, and an embedded hyperlink to the web-based survey (via Survey Monkey). Descriptions of the constructs (occupational stress and EE) were operationally defined in the instructional section of the invitation letter. Non-DCs and/or DCs that were not involved in chiropractic fieldwork were identified and excluded from the study via two qualification questions. The remaining participants were asked (in open-ended format) to describe the occupational stressor(s) - if any - that they believed had a negative impact on their professional and personal life. The open-ended question (dependent variable) read as such: ‘*What factors do you feel influence the levels of occupational stress and emotional exhaustion in the chiropractic profession?’*

A mixed methods approach was used to explore respondents’ perceptions of occupational stress and EE. The qualitative portion of this research design used content analysis [25, 26], inductively, coupled with an epistemological assumption(s) and interpretational approach, as the foundation of analysis to the open ended responses. Moreover, the qualitative analytic strategy employed in this study relied on a general approach that involved interpretive description as a means of developing an understanding of occupational stress and EE endured by DCs. The aim was to generate categories of reason for occupational stress and EE by using content analysis [27, 28]. Each open-ended response was read thoroughly and organized into categories of reason. If the response included two or more different statements of reason – it was identified and categorized accordingly to form the separate responses. For example, a typical response like “…*low fees, too much paperwork, too much government regulation, deny-minded IME interfering with care, staff training, high expenses*” includes many items that could fall in one (Business & Administration) or more (MCO regulation and reimbursement) categories. Categories were not preconceived and were named using respondents’ own terminology where possible. The principle investigator (PI) read all of the statements in the initial sample and carried out this analysis on two additional separate occasions. The initial analysis generated almost identical sets of categories. A set of 14 categories was composed based directly on the respondents’ statements.

The quantitative analysis integrated summaries of the categorical themes that were obtained after the open-ended responses, with similar themes grouped together. Descriptive statistics involving the frequency and percentage summaries were conducted to determine the number of respondents that chose each of the categorical themes. Cross tabulation(s) were conducted to match the demographic data of gender, age, years in practice, marital status, current professional status, and location of practice with the categorical themes of the open-ended responses. Chi square statistical test(s) were conducted to determine if the demographic data was significantly related to, or differed with, the categorical themes of the open-ended responses. A level of significance of 0.05 was used in the statistical analysis. During the study, several methods were used to ensure the data trustworthiness (i.e. practices supporting credibility, transferability, dependability, confirmability) as outlined by Zhang [25]. All analyzes were conducted in SPSS. Ethics approval for the study was obtained by Seton Hall University’s IRB in January 2013.

## Results

### Descriptive statistics

Most of the respondents were solo-practitioners and practicing in the United States. Additionally, most of the respondents were male and were married. It was observed that the age of those that answered the open-ended questions were between 31 and 60 years old.

The open-ended question was examined for common themes. The most-common responses were Managed Care Organization (MCO) Regulation (33 %), MCO Reimbursement (26.8 %), and Scope of Practice Issues (21.3 %). There were also significant numbers of responses for Business and Administrative (16.4 %), Public Perception / Public Acceptance (16.1 %), Self–Perception / Purpose (11.2 %), and Economy / Money (10.3 %).

In the 14 categorical themes of response, it was observed that many of the respondents practiced chiropractic in California, Colorado, Connecticut, Florida, Georgia, Illinois, New Jersey, New York, North Carolina, Ohio, Pennsylvania, and Texas. The percentage distribution of gender, the number of years in the professions and the practice location found in this study was reflective of current industry data [29, 30].

### Qualitative – content analysis

There were a total of 970 analyzed open-ended responses out of the 1149 total respondents (Table 1); thus resulting in an 84 % completing rate. Those remaining 179 surveys were deemed incomplete. The initial analysis of 2022 statements generated 14 subcategories that collectively described perceptions and/or potential sources of occupational stress and EE. A further grouping of these subcategories was executed reflecting the context of the healthcare system, at-large. This stage of analysis produced three main categories, which is also described in the literature as common, conventional, and cultural specific [27, 31]. For the purpose of understanding the causes of occupational stress in DCs the three main categories were observed as (1) Health Care System - Conventional, (2) Intra-professional conflict- Cultural Specific, (3) Personal / Individual attributes - Common. Table 2 represents the frequencies and a percentage for all subcategories found, and just equally as important reflects, the magnitude / impact of DCs perception(s) of occupational stress.

**Table 1:** Descriptive frequencies for open-ended factors

		Answered open ended response
Gender	Female	179
	Male	791
Total		970
Age	20–30	47
	31–40	177
	41–50	267
	51–60	329
	61–70	128
	70+	22
Total		970
Years of practice	0–5	70
	6–10	100
	11–15	168
	16–20	118
	20–25	143
	25–30	154
	30+	217
Total		970
Marital status	Married	784
	Widowed	6
	Divorced	90
	Separated	11
	Never married	79
Total		970
Current professional status	Associate	46
	Independent contractor	42
	Sole-Practitioner	651
	Group practice	197
	Not a direct care provider (academic and/or administrative)	34
Total		970
State or U.S. territory practicing chiropractic	Australia or New Zealand	8
	Canada	31
	UK & Europe	10
	United States	672
Total		721

**Table 2:** Frequencies and percentages for open-ended factors

Factor	Number	Percent
Business and Administrative	190	16.4
MCO Reimbursement	311	26.8
Isolation	26	2.2
Lack of vacation	24	2.1
MCO Regulation	384	33
Intra-Professional Stress	151	13
Patients	112	9.6
Public Perception / Public Acceptance	187	16.1
Self–Perception / Purpose	130	11.2
Student loan debt	35	3
Economy / Money	120	10.3
Scope of Practice Issues	247	21.3
Physical demands	28	2.4
Working too hard (long hours)	77	6.6

Percentages may not total 100 due to rounding error

#### Conventional: deficiencies of the health care system

The statements expressing external sources of stress with the way health care services are organized were further compartmentalized into respective subcategories. One of the assumptions of such categorization is that many similar helping professions share similar sources of occupational stress and EE. Globally, these statements reflect the respondents’ dissatisfaction with the perceived dysfunction of the MCOs including perceived problems with regulation of the health care system, legislation and implementation of it or cost for services. These reasons were grouped into major distinctive groups, such as: MCO regulation and MCO reimbursement.

*‘Dealing with Insurance, third party administrators, insurance reviews.’*

*‘Constant changes and dealing with/arguing with insurance companies. It’s a constant fight to maintain a sustainable income due to constant decreases in reimbursement.’*

*‘Uncertainty regarding the future of healthcare law and policy. Unfair reimbursement through the third party payer system and Medicare program.’*

#### Cultural-specific: deficiencies of the chiropractic profession

The statement expressing intra-professional sources of stress and exhaustion with the way the chiropractic profession is organized. Globally, these statements reflect the respondents’ sources of stress with the chiropractic profession as a unit. Further, the statements describe problems that appear to be unique to the chiropractic profession and/or may be reflective of the occupational stressors that other alternative medicine professions, at-large, experience. These reasons were grouped into distinctive groups: Opposing DC views, Public Acceptation/Perception, Scope of practice issues

*‘The lack of cultural authority along with divergence in clinical practice.’*

*‘Persistent negative stereotypes/perception in the general public/media. Constant infighting within the profession. Atmosphere of competition among peer chiropractors, including dishonesty regarding the state of one’s practice in talking with other chiropractors.’*

*‘The insurance companies treating us as less than doctors and making our lives miserable by questioning our care. I want to help patients get well not spend all day on paperwork.’*

*‘Lack of cultural authority, lack of solidarity, the fact that DC’s don’t have a common answer for what we do, too much infighting, lack of professionalism in the profession, allowing practice building groups taking advantage young practitioners.’*

#### Deficiencies of the DCs attitudes, skills and work

These statements described perceived deficiencies of individual practitioners. Globally, the statements describe basically all aspects of practice: knowledge, skill, behaviors and attitudes. These reasons were grouped into distinctive groups: Self-Perception, Isolation, Working too hard and Business and Administrative.

*‘Having no goals, having a poor vision for the future and not willing to grow. Also, a long term view is necessary with goals to match and some risk taking make practice more interesting and exciting.’*

*‘Unrealistic expectations upon entering profession. Inadequate communication skills. Poor business operation skills.’*

### Quantitative analysis

#### Cross tabulations

Both male and female DCs believed that the top two stressors that negatively influence their professional work were MCO Regulations (male 303; female 81) and MCO Reimbursement (male 252; female 59). Scope of Practice Issues (male 204; female 43) and Public Perception / Public Acceptance (male 143; female 44) and Business and Administrative (male 153; female 37) were also noted as significant stressors across both genders. The same trend was observed in those DCs that were currently married. The age of the participants and the number of years in their profession was spread fairly evenly. When investigating the top three stressors that influence their professional lives, those DCs aged 31 to 40, 41 to 50, and 51 to 60 years old respectively, believed that MCO Regulations (65, 101, 144), MCO Reimbursement (61, 89, 111), and Scope of Practice (49, 58, 85) had the most noteworthy negative impact. Interestingly, DCs that said MCO Regulations, MCO Reimbursement, and Scope of Practice Issues were the most influential stressors in practice – were also those that had most years in active practice (20 -30+ years in practice). Of the 311 respondents that noted MCO Reimbursement as a significant stressor, 208 were sole-practitioners and 69 were in group practice. Of the 384 respondents that noted MCO Regulations as a significant professional stressor, 262 were sole-practitioners and 74 were in group practice. Of the 247 DCs that noted Scope of Practice, 167 were sole-practitioners and 48 were in group practice.

Collectively, when investigating the top three stressors that influence their professional lives, DCs noted MCO Regulations, MCO Reimbursement, and Scope of Practice Issues, the State or U.S. territory the respondents were practicing chiropractic was mostly from California, New York, and Pennsylvania. Of the 311 respondents that noted MCO Reimbursement, 37 were from Pennsylvania, 35 from California, and 33 from New York. Of the 384 respondents that noted MCO Regulations, 51 were from Pennsylvania, 41 from New York, and 35 from California. For the 247 respondents that noted Scope of Practice Issues, 33 were from California, 18 from New York, and 12 from Pennsylvania.

#### Chi-square test results

The results of the chi-square test showed that gender was significantly related with the responses of MCO Regulation (X^2^ (1) = 3.93, *p* = 0.05), Patients (X^2^ (1) = 35.35, *p* = 0.02), Public Perception / Public Acceptance (X^2^ (1) = 4.76, *p* = 0.03), and Working too hard (X^2^ (1) = 6.35, *p* = 0.01). Chi-square test also showed that age was significantly related with the responses of business and administrative (X^2^ (5) = 11.68, *p* = 0.04), patients (X^2^ (5) = 18.88, *p* <0.01), self-perception (X^2^ (5) = 15.99, *p* = 0.01), and working too hard (X^2^ (5) = 16.90, *p* = 0.01). Additionally, chi-square test also showed that the number of years in practice was significantly related with the responses of MCO regulation (X^2^ (6) = 12.68, *p* = 0.05), patients (X^2^ (6) = 27.07, *p* <0.001), other (X^2^ (6) = 15.12, *p* = 0.02), and working too hard (X^2^ (6) = 22.18, *p* <0.001); and that marital status was significantly related with the responses of patients (X^2^ (4) = 17.72, *p* <0.001), economy (X^2^ (4) = 13.04, *p* = 0.01), and working too hard (X^2^ (4) = 11.60, *p* = 0.02). Collectively, this indicates that the two gender groups, the six age groups, the seven categories of years in practice and the five categories of marital status have significant different responses in the open-ended responses. As per the various DC working characteristics, the results of the chi-square test showed that current professional status was significantly related with the responses of Intra-Professional Stress (X^2^ (4) = 14.94, *p* = 0.01), Patients (X^2^ (4) = 26.11, *p* <0.001), and Student Loan Debt (X^2^ (4) = 12.40, *p* = 0.02). Additionally, location of practice (Table 3) was significantly related with the responses of insurance reimbursement (*X*^2^ (3) = 9.77, *p* = 0.02), MCO regulation (X^2^ (3) = 13.44, *p* <0.001), and self-perception (X^2^(3) = 9.89, *p* = 0.02). Collectively, this indicated that the five categories of current professional status and the four categories of location of practice have significant different responses in the open-ended responses of Intra-Professional Stress, Patients, and Student Loan Debt, and MCO Reimbursement, MCO Regulation, and Self-Perception / Purpose, respectively.

**Table 3:** Chi-square result of relationship between location of practice and categorical themes of response

	Value	df	Asymp. Sig. (2-sided)
Business and administrative	3.22	3	0.36
Insurance reimbursement	9.77	3	0.02^a^
Isolation	0.44	3	0.93
Lack of vacation	1.50	3	0.68
MCO regulation	13.44	3	0.00^a^
Opposing DC views (Identity) Intra-Professional Stress	4.48	3	0.21
Patients	1.31	3	0.73
Public perception (cultural authority)/ public acceptance	5.54	3	0.14
Self-perception (purpose)	9.89	3	0.02^a^
Student loan debt	0.78	3	0.86
Economy (money)	0.09	3	0.99
Other (scope)	1.67	3	0.64
Physical demands	1.50	3	0.68
Working too hard (long hours)	1.79	3	0.62

^a^Significant relationship at Level of Significance of 0.05

## Discussion

The primary aim of the current study was to examine the perceptions of occupational stress among a representative sample of chiropractors in the US. This mixed methods approach, with emphasis on the qualitative analysis, generated three main categories and 14 subcategories representing the perceived occupational stressors among DCs. Overall, the results showed that the most of the participants believed that MCO regulation, MCO reimbursement, and Scope of practice issues were the most common stressors that negatively influenced their professional and personal lives. Interestingly, scope of practice amongst DCs is highly variable [32–34] in the US, and when coupled with cost of living differences, a strong connection between these factors became apparent. The participants responses indicated their perception of a cause effect relationship between occupational stress, emotional exhaustion and “*cultural authority, government / Obama, education, long hours, time, tools, medical, competition for other professions, documentation, scope, expectations; overhead; risk; scope of practice; paperwork; State associations; college / school; unethical; pay; EHR/EMR; communicating; balance; respect; unity; reward; AMA; boredom”*. High student loans, the non-recognition by the medical community, and the administrative aspects of operating a business, also have a significant negative implication(s) on DCs’ practice life; by means of reducing resources and increase demands, as outlined in the control-stress model [5] and job-demands control model [10, 11]. However, collectively it appeared that most significant stressor within the chiropractic profession is “*the frustration with the insurance companies*”. Complaints of constantly getting denied, the extremely low reimbursement (gets lower every year), the raising of co-pays to make patients not want to come in appear to be overwhelming the modern day DC. These findings are consistent with much of the current occupational stress research [10, 20–22, 27]; which lends the notion that major changes in the health care system have been driven by increase-regulation via third-party payer systems.

Similar precursors/processes to occupation stress and EE have been observed in a comprehensive group of health professionals [2, 4, 17, 35–37] – and while some stressors were consistent across occupations, others were more rare or occupation specific. Across health professions, it appears that healthcare workers suffer from occupational stress because of higher expectations, not enough time, lack of skills and social support at work [21, 35–37]. Notably, interpersonal conflict appears to be the most prevalent stressor across all occupations [20, 22] – organizational constraints and workload are just as commonly reported in the literature. Interpersonal conflict occurs when a person or group of people frustrates or interferes with another person’s efforts at achieving a goal [38] – and may be reflective of the unique cultural-specific perceptions of stress that occur in the chiropractic profession.

As the content analysis progressed, a conceptual pattern amongst the participants began to unfold. It appears that many of the participants agreed – Chiropractic’s lack of internal consensus and legitimacy (cultural authority) inhibits chiropractic’s ability to keep up with rapidly changing events. Further, participants repeatedly noted/suggested that in order for the chiropractic profession to progress, that is keep up with external health care events, e.g., the Affordable Care Act, Health Care Education Reform Act, etc., the profession would needs to come to some modicum of internal consensus. Internal consensus will be needed if the profession is going to achieve cultural authority [39]. Keeping the profession rooted in metaphors — i.e., Universal Intelligence, Innate Intelligence, Subluxation, dis-ease, etc. — which for some have become unquestionable myths and dogma inhibits chiropractic’s achievement of legitimacy, the other necessary ingredient of cultural authority [40].

### Limitation of the analysis

The limitation of the categorical analysis involving determining the relationship between the demographics with the categorical themes of open-ended responses is that causality cannot be determined. Also, finding a significant relationship between two variables with a correlation coefficient does not take into account the possibility of other variables playing a part. In addition, the direction (positive or negative) of the relationship and the strength of the relationship (weak, moderate, and strong) cannot be determined with a chi-square test. The variables of demographic and categorical themes of open-ended responses are categorical variables. Thus, correlation test cannot be conducted. The analysis merely determined the relationship between variables by investigating whether there is significance different in the categorical responses according to the results of the chi-square analysis.

## Conclusion

The findings from this current study add to a continuous dialog on the unique causes of stress, emotional exhaustion and occupational stress for chiropractic professionals. These findings in this study add to the notion of directional association between third party payer influences (increased regulation/decreased reimbursement) with that of increased job stress. Further research will be undertaken to refine the stress and satisfaction parameters and address stress interventions.
